# New injectable two-step forming hydrogel for delivery of bioactive substances in tissue regeneration

**DOI:** 10.1093/rb/rbz018

**Published:** 2019-05-10

**Authors:** Edgar Pérez-Herrero, Patricia García-García, Jaime Gómez-Morales, Matias Llabrés, Araceli Delgado, Carmen Évora

**Affiliations:** 1 Department of Chemical Engineering and Pharmaceutical Technology, University of La Laguna, La Laguna, Tenerife, Spain; 2 Institute of Biomedical Technologies (ITB), Center for Biomedical Research of the Canary Islands (CIBICAN), University of La Laguna, La Laguna, Tenerife, Spain; 3 Laboratory of Crystallographic Studies, Andalusian Earth Sciences Institute, Spanish Research Council—University of Granada, Armilla, Granada, Spain; 4 Institute of Tropical Diseases and Healthcare of the Canary Islands, University of La Laguna, La Laguna, Tenerife, Spain

**Keywords:** hydrogel, collagen–cyclodextrin–chitosan, rheology, mass transfer, estradiol, FITC-dextran

## Abstract

A hydrogel based on chitosan, collagen, hydroxypropyl-γ-cyclodextrin and polyethylene glycol was developed and characterized. The incorporation of nano-hydroxyapatite and pre-encapsulated hydrophobic/hydrophilic model drugs diminished the porosity of hydrogel from 81.62 ± 2.25% to 69.98 ± 3.07%. Interactions between components of hydrogel, demonstrated by FTIR spectroscopy and rheology, generated a network that was able to trap bioactive components and delay the burst delivery. The thixotropic behavior of hydrogel provided adaptability to facilitate its implantation in a minimally invasive way. Release profiles from microspheres included or not in hydrogel revealed a two-phase behavior with a burst- and a controlled-release period. The same release rate for microspheres included or not in the hydrogel in the controlled-release period demonstrated that mass transfer process was controlled by internal diffusion. Effective diffusion coefficients, *D*_eff_, that describe internal diffusion inside microspheres, and mass transfer coefficients, *h*, i.e. the contribution of hydrogel to mass transfer, were determined using ‘genetic algorithms’, obtaining values between 2.64·10^−15^ and 6.67·10^−15^ m^2^/s for *D*_eff_ and 8.50·10^−10^ to 3.04·10^−9^ m/s for *h*. The proposed model fits experimental data, obtaining an *R*^2^-value ranged between 95.41 and 98.87%. *In vitro* culture of mesenchymal stem cells in hydrogel showed no manifestations of intolerance or toxicity, observing an intense proliferation of the cells after 7 days, being most of the scaffold surface occupied by living cells.

## Introduction

Hydrogels are three-dimensional hydrophilic polymeric networks, which are able to absorb and retain large quantities of water, solvents or biological fluids in the free space of their structure without being dissolved in the same media. They can be generated through physical crosslinking by weak cohesive forces, like ionic or hydrogen bonds and π–π or van der Waals interactions, or chemical crosslinking by stable covalent forces that improve the mechanical properties of the hydrogel. However, the toxicity derived from the use of crosslinking agents, like glutaraldehyde, and the possibility of irreversible broken bonds that can fracture the structure, are some of the drawbacks of the chemical crosslinking [1–3]. The high permeability and porosity of hydrogels facilitates, not only integration in the majority of tissues, but also incorporation by the cells, and their biocompatibility and biodegradation avoid surgery to remove polymer after treatment [2, 3]. Moreover, their similarity to the extracellular matrix or tissues, low interfacial tension between gel and solvents, and minimal irritation after application due to their water content and soft nature [2, 3] have made hydrogels the ideal candidates for biomedical and pharmaceutical applications, such as implants and sutures, wound dressings, controlled drug delivery, diagnostic imaging, medical and biological sensors or microfluidics [3, 4]. In this regard, *‘in situ’* two-step forming injectable hydrogels have attracted the attention of scientific community since they can be implanted without surgery directly by injection in the treatment site, where the crosslinker agent forms stable scaffolds. Rheology of this type of hydrogels provides adaptability to the shape of the area to be treated, an easy ‘*in vivo*’ implantation in a minimally invasive way and capacity to incorporate bioactive substances and/or cells.

A long list of biodegradable natural or synthetic polymers has been used to prepare hydrogels. Natural polymers include polysaccharide-based polymers (chitosan, alginate, cellulose, dextran, gellan or guar gum, cyclodextrins and hyaluronic acid) and protein-based polymers (gelatin, collagen, albumin, fibrin or heparin). Although they are abundant in nature and can be part of biological events, such as signaling or cell adhesion, they do not have good mechanical properties and tunable degradation by themselves and can lead to possible immunogenic responses [4, 5]. In particular, polysaccharide-based hydrogels offer interesting options to enclose molecules since they can be easily formed by mild conditions methods, like ionic gelation or complexation in aqueous environments at room temperature, avoiding the use of organic solvents and high shear homogenization or ultrasonication. In fact, chitosan, a cationic natural linear polymer derived from chitin and composed of d-glucosamine and *N-*acetyl-d-glucosamine units, gels by interaction with polyanions, like the negatively charged and nontoxic tripolyphosphate (TPP) [6]. Chitosan-based hydrogels have been used as injectable platforms in controlled drug delivery and tissue engineering applications [5]. Moreover, chitosan can be mixed with the protein-based polymer collagen since both solutions are miscible and chitosan does not denature collagen [7]. The strategy of adding collagen to hydrogel formulation brings advantages like enhanced mechanical strength, biocompatibility and biodegradability, and a more efficient cellular growth [4]. Although collagen can form gels by natural means, the resulting mechanical strength is weak, being necessary the use of crosslinking agents. For example, glutaraldehyde or formaldehyde have demonstrated effectiveness in creating crosslinking structures in collagen but limit their application in biomedical systems because of their cytotoxicity [3, 8]. Another option to crosslink collagen is the use of water-soluble carbodiimides, although they are not usable when the enclosed bioactive substances are proteins because they react with them [3]. Riboflavin combined with ultraviolet-A light has been widely used in clinic to reinforce the collagen structure of the cornea in the treatment of keratoconus [9, 10]. Therefore, this seems to be a good strategy to induce crosslinking of collagen and increase its mechanical strength without compromising cell viability. Moreover, the addition of cyclodextrins, a cyclic oligosaccharide composed of six to eight glucopyranose units, can lead to the formation of inclusion complexes with certain polymer regions. The toxicity of these compounds decreases with increasing number of glucopyranose units, thus, gamma cyclodextrins are less toxic than alpha or beta ones [11]. Synthetic polymers, like poly(dl-lactide-co-glycolide) (PLGA), poly(dl-lactide) (PLA), poly(vinyl alcohol) (PVA), poly(acrylic acid) (PAA), polyethylene glycol (PEG), can enhance and control the mechanical strength and degradation rates of hydrogels. Still, they do not provide the optimal environment for cell inclusion and tissue regeneration, so, a combination of both types of polymers, natural and synthetic, must be used for the biomedical application of hydrogels [5, 12, 13]. Moreover, the incorporation of inorganic components into the hydrogel structure, such as hydroxyapatite (HAp) i.e. part of the bone matrix, have been used in hard tissue repair to reinforce the mechanical properties of the hydrogel [14]. In this regard, nanosized HAp (nano-HAp) should be used to promote their degradation by osteoblastic enzymes, like the alkaline phosphatase, and form new tissue by osteoblasts, being this size the optimum for enhanced cell adhesion and proliferation [14].

Bioactive substances can be released from hydrogels by either degradation of polymer or diffusion through the pores of their structure, or by a combination of both. Release profiles in these systems typically reveal a two-phase behavior, a burst step followed by a slow controlled release period. Because of burst may cause high concentrations of bioactive substances in the application site and consequently loss of these drugs, more complex systems are required to reduce such burst profiles, maintaining the total delivery values in longer periods.

In this work, an innovative hydrogel based on biocompatible and biodegradable polymers, such as, chitosan, collagen, hydroxypropyl-γ-cyclodextrin (HP-γ-CD) and PEG, has been designed, developed and characterized in terms of porosity, rheology and mass transfer studies. The hydrogel was injectable, adaptable to treatment sites with diverse dimensions and shapes, easily crosslinked by means of TPP and blue light and presented adequate characteristic for good cell adhesion, viability and proliferation. Moreover, 17-β-estradiol or rhodamine-B-isothiocyanate-dextran (RITC-dextran), as low molecular weight hydrophobic or high molecular weight hydrophilic model drugs, respectively, were encapsulated in PLGA/PLA microspheres and included in the hydrogel, together with the nano-HAp for further use in bone regeneration.

## Materials and methods

### Materials

PLGA Resomer^®^ RG504 (acid-terminated, lactide:glycolide 50:50, M_w_ 38–54 kDa) and RG858S (ester-terminated, lactide:glycolide 85:15, M_w_ 190–240 kDa) and PLA Resomer^®^ RG203-S (ester-terminated, M_w_ 18–28 kDa) were acquired from Boehringer-Ingelheim (Germany). HP-γ-CD (CAVASOL^®^ W8 HP) was obtained from Wacker (Germany). Ultrapure chitosan Protasan^TM^ UP-CL-213 (86% deacetylation, viscosity 150 mPa·s at 1 wt. % aqueous solution) and ultrapure alginate Pronova^TM^ UP MVG were purchased from NovaMatrix (Norway). Bovine collagen, type I, was purchased from CellSystems Biotechnologie Vertrieb GmbH (Germany). Calcium chloride dihydrate (Bioxtra, ≥99% pure), sodium citrate tribasic dihydrate (ACS reagent, ≥99% pure), disodium hydrogen phosphate (ACS reagent, ≥99% pure), sodium carbonate monohydrate (ACS reagent, 99.5% pure), hydrochloric acid (ACS reagent, 37 wt. % in H_2_O), 17β-estradiol, dichloromethane (puriss ≥99, 0% pure), PVA (Mw 30–70 kDa, 87–90% hydrolyzed), riboflavin (≥98% pure), PEG 400 (for synthesis), RITC-dextran (average M_w_ 70 kDa) and TPP (practical grade, 90–95% pure) were provided from Sigma Aldrich (St. Louis, MO, USA). All solutions were prepared with ultrapure water (0.22 μS, 25°C, Milli-Q, Millipore). High glucose DMEM and penicillin/streptomycin were acquired to HyClone (Utah, USA) and PAA laboratories (Pashing, Austria), respectively. Fetal bovine serum (South America Origin) and l-glutamine were provided from Biowest (France). Calcein-AM and *p*-formaldehyde were purchased from Fluka Analytical (USA) and Panreac (Spain), respectively.

### Microspheres preparation

RITC-dextran-loaded PLGA microspheres were prepared, as previously described [15], by double emulsion solvent evaporation method (water/oil/water). Briefly, a first emulsion was formed by mixing (vortex) 200 µl of a solution of 14.4 mg/ml of RITC-dextran (2880 µg) in 0.2% w/v PVA with 1 ml of DCM containing 150 mg of PLGA [RG504: RG858S (4:1)]. Then, this first emulsion was poured into 10 ml of 0.2% w/v PVA under vortex, forming the second emulsion that was added over 100 ml of 0.1% w/v PVA under magnetic stirring. This preparation was then left for 1 h under mild agitation to evaporate the solvent. Microspheres were collected by filtration, lyophilized and stored at 4°C until required. Blank microspheres were prepared with the same method but using as aqueous phase 200 µl of a solution of 0.2% w/v PVA without RITC-dextran.

17-β-Estradiol-loaded PLA/PLGA microspheres were prepared by simple emulsion solvent evaporation method (oil/water). Briefly, 0.6 ml of an organic phase DCM: MeOH (83:17) containing 200 mg of polymer PLA/PLGA [RG-203S: RG858S (4:1)] and 4 mg of 17-β-estradiol were mixed by vortex with 4 ml of 1% w/v PVA to form an emulsion that was poured into 96 ml of 0.16% w/v PVA under magnetic stirring. Then, the formulation was left 1 h under mild agitation to allow the evaporation of the solvent. Microspheres were collected by filtration, lyophilized and stored at 4°C until use. Blank microspheres were synthetized using the same method but removing 17-β-estradiol from the organic phase.

### Carbonated apatite nanoparticles preparation (nano-hydroxyapatite)

Citrate-coated carbonated apatite nanoparticles were prepared by the thermal decomplexing of metastable solution containing Ca^2+^–citrate complexes in the presence of phosphate and carbonate ions at pH = 8.5 [16, 17]. Powders were synthesized as follows: 50 ml of a solution (a) of composition 0.06 M Na_2_HPO_4_ + 0.1 M Na_2_CO_3_ was poured into 50 ml of a solution (b) of composition 0.1 M CaCl_2_ + 0.2 M Na_3_(cit) at 4°C. Then, pH was adjusted to 8.5 with diluted HCl. The mixed solution was introduced into a bottle sealed with a screw cap, immediately submerged in a water bath at 80°C and then moved to an oven with circulated forced air at the same temperature. The experiments lasted 96 h. Upon completion, the precipitates were washed with ultrapure water by six consecutive cycles of centrifugation to remove unreacted species or salts. Afterward they were freeze-dried overnight. Theoretical reaction yielding carbonated apatite nanoparticles can be represented by:

$$\text{5 Ca}{\left(\text{cit}\right)}^{-}\;+\;{\text{3HPO}}_{\text{4}}{}^{-}\;+\;\left(x\;+\;y\right){\text{ CO}}_{\text{3}}{}^{\text{2}-}\;+\;{\text{OH}}^{-}\to \;{\text{Ca}}_{\text{5}}{\left(\text{OH}\right)}_{\text{1}-x}{\left({\text{CO}}_{\text{3}}\right)}_{x}{\left({\text{PO}}_{\text{4}}\right)}_{\text{3}-y}{\left({\text{CO}}_{\text{3}}\right)}_{y}\;+\;{\text{5cit}}^{\text{3}-}\;+\;{\text{3H}}^{+}\;+\;x{\text{OH}}^{-}$$

### Hydrogel preparation

A 10 mg/ml type I collagen solution in a cold solution of acetic acid 0.043 M, a 0.43 g/ml aqueous solution of HP-γ-CD with riboflavin (5 mg/ml) and a 20 mg/ml solution of chitosan in water were prepared. In addition, pure PEG 400 (density: 1.128 g/ml) was used. Hydrogel was prepared by mixing with vortex the enough quantity of all the previous components to obtain the following final concentrations, 5.3 mg/ml of collagen, 33.8 mg/ml of HP-γ-CD (0.4 mg/ml of riboflavin), 5.3 mg/ml of chitosan and 148.4 mg/ml of PEG 400. Afterward, this hydrogel was crosslinked with 25 µl of 5% w/v TPP sterile aqueous solution per 50 µl of hydrogel under blue visible light (468 nm, quartz-tungsten halogen Hilux UltraPlus, Benlioglu Dental Inc.). In addition, hydrogels with microspheres and nano-HAp were prepared by adding 14 mg of blank or 17-β-estradiol-loaded PLA/PLGA microspheres, 5 mg of blank or RIBD-dextran-loaded PLGA microspheres and 5 mg of nano-HAp per 50 µl of hydrogel, simulating the therapeutic doses of 17-β-estradiol and osteogenic proteins, such as BMP-2, in 50 µl of hydrogel.

### Characterization of microspheres, nano-HAp and hydrogels

Size and morphology of microspheres were determined by laser diffractometry (Mastersizer 2000, Malvern Instruments) and scanning electron microscopy (SEM, Jeol JSM-6300), respectively.

Size and morphology of nano-HAp were performed by transmission electron microscopy (TEM) with a Carl Zeiss Libra 120. The composition analysis of the carbonated apatite nanoparticles were carried out by X-ray diffraction (XRD) and Fourier transform infrared (FTIR) spectroscopy. XRD data were collected by using a Bruker D8 Advance Vario diffractometer with Bragg–Brentano parafocusing geometry. FTIR spectrum of the sample was recorded in transmittance mode with a Perkin-Elmer Spectrum One FTIR spectrometer within the wavenumber range from 4000 to 400 cm^−1^ at a resolution of 4 cm^−1^, using a pellet that was prepared by mixing ∼1 mg of sample with 100 mg of anhydrous KBr and then pressed with a hydraulic pump into 13 mm diameter discs.

Porosity of hydrogels, with and without microspheres and nano-HAp, was calculated from the true density (helium pycnometer, Micromeritics AccuPyc 1330) and the apparent density of the freeze-dried hydrogels prepared in a graduated cylindrical mold. To avoid collapse processes during freeze-drying, the hydrogel was maintained in a mold and quickly frozen with liquid nitrogen before being exposed to a high-vacuum lyophilization process. The existence of true pores was verified by means of SEM.

Viscoelastic and thixotropic behaviors of hydrogel were obtained with a Bohlin CVOD 100 rheometer at 37°C by means of a Peltier system, using cone-plate and parallel geometries with a diameter for the fixed lower plate of 60 mm and a gap between the fixed and rotating part of 1 mm. The evolution of viscosity with shear rate (from 0.071 to 30 s^−1^) was acquired by cone-plate geometry (diameter of cone 40 mm, angle 4°). The evolution of elastic (G’) and viscous moduli (G’’) with frequency (from 0.128 to 4.015 Hz) was acquired by a parallel plate geometry (diameter of rotating upper plate 20 mm) at a constant shear stress of 0.2387 Pa. Note that for the viscoelastic characterization, the final crosslinking step with blue light and 5% w/w TPP was not used and only blank microspheres were used.

Analysis of the functional groups of the components of the hydrogel were determined by FTIR spectroscopy with a Bruker IFS 66/S, in transmittance mode, within the wavenumber range from 4000 to 400 cm^−1^ at a resolution of 4 cm^−1^, using the attenuated total reflectance accessory that permit to examine samples in the solid state.

*In vitro* release profiles and encapsulation efficiencies of 17-β-estradiol and RITC-dextran from microspheres and microspheres-loaded hydrogels were determined at 37°C and 120 rpm (orbital agitator) with a spectrophotometer (Ultrospec 3300 ‘pro’, Amersham Biosciences) at 280 or 555 nm, respectively. Precisely, 26 mg of 17-β-estradiol-loaded PLA/PLGA microspheres (382 µg), included or not into 100 µl of hydrogel were placed in a vessel with 4 ml of release medium. Simultaneously, 70 mg of RITC-dextran-loaded PLGA microspheres (1071 µg) included or not into 200 µl of hydrogel were placed in a vessel with 2.5 ml of release medium. The release media consisted of 0.9% NaCl solution, which was mixed with methanol in a ratio 60:40 to increase the solubility of the hydrophobic drug and then assess the sink condition, or ultrapure water. Two methods of sampling were used, 500 µl of media was collected at different time points and then either returned to the vessel (batch) or removed and replaced with fresh medium (semi-continuous).

All the experiments performed to characterize the microspheres and the hydrogel were performed in the SEGAI (Servicio General de Apoyo a la Investigación) of the Universidad de La Laguna.

### Cell culture

#### Cell isolation

All animal experiments were performed according to the EC directive 2010/63/EU on Care and Use of Animals in Experimental Procedures. Furthermore, animal experiments were approved by the committee for animal studies of Universidad de La Laguna. Mesenchymal stem cells from femur bone marrow of 6 weeks-old male Sprague Dawley rats (rMSC) were obtained by centrifugal isolation as previously described by Dobson *et al*. [18]. Briefly, the cells were resuspended in high glucose DMEM supplemented with 10% fetal bovine serum, 1% penicillin/streptomycin and 2 mM l-glutamine stable (complete culture medium, CCM). They were seeded in flasks of 75 cm^2^ and incubated at 37°C and 5% CO_2_. Every 2–3 days the culture medium was changed until ∼80% of confluence.

#### Culture of rMSC in the hydrogel and cell viability

Aliquots of 300 µl of crosslinked collagen gel were placed into a 48-well plate. Then, 50 000 cells in 20 µl of CCM were added to each well and incubated at 37°C and 5% CO_2_ for 1.5 h for cell adhesion. The homogeneous cell distribution was confirmed by light microscopy. Afterward, 500 µl of CCM were added to each well, and the plates were incubated during 7 days and the medium changed every 3 days.

The seeded cell viability was tested with calcein-AM. After 1, 5 h and 1, 3 and 7 days of culture, 3 wells of each time were washed (3 times) with Hank’s balanced salt solution (HBSS 1×). Then 500 µl of 1 µM calcein-AM in HBSS solution were added and incubated at 37°C and 5% CO_2_ under soft agitation for 30 min. Then, calcein was removed and cells were fixed with a solution of 3.7–4% *p*-formaldehyde buffered to pH = 7.0 during 30 min. After this, formaldehyde was removed, and the wells were washed 3 times with HBSS 1×. Immediately after this, viable cells were visualized using a fluorescence inverted microscope (EVOS FL, Life Technology, Invitrogen).

## Results and discussion

### Nano-HAp characterization

The nanoparticles displayed plate-shape morphologies with average length (L = 40.0 ± 5.0 nm) and width (W = 9.5 ± 1.2 nm) (TEM).

**Figure 1:**
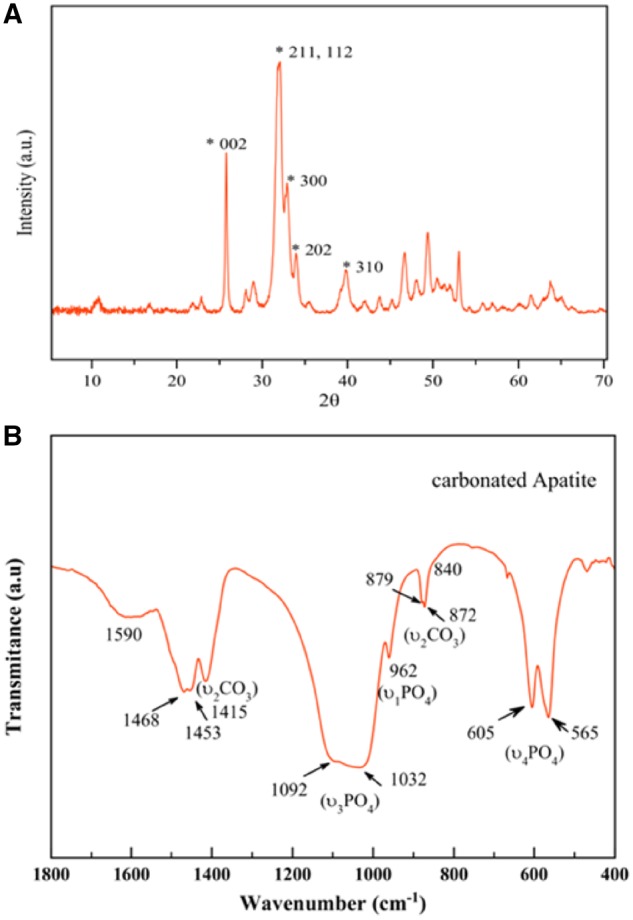
(A) XRD pattern of carbonated apatite prepared by thermal decomplexing of Ca/citrate/phosphate/carbonated solutions at pH = 8.5 at 80°C. (B) FTIR spectrum in transmittance mode of carbonated apatite prepared by thermal decomplexing of Ca/citrate/phosphate/carbonated solutions at pH = 8.5 at 80°C

The XRD pattern of the sample is reported on Fig. 1A and correspond to the apatite phase (PDF 01-1008), with peaks at 2θ = 25.87° corresponding to the (002) plane, the triplet at 31.77°, 32.19° and 32.90° (planes (211), (112) and (300)) respectively, the reflections at 33.9° and 39.81° (planes (202) and 310)) and other minor peaks in the 2θ range from 40^°^ to 55°. The absence of octacalcium phosphate (OCP) is witnessed by the absence of the main reflection at 4.74°, plane (100), (OCP, PDF 44-0778).

Figure 1B shows the FTIR spectrum of the sample in 400–1800 cm^−1^ region. The main band at 1000–1100 cm^−1^ corresponds to the asymmetric stretching mode of ${\text{PO}}_{4}^{3-}$ groups (υ_3_ PO_4_). The shoulder at ∼965 cm^−1^ is ascribed to the symmetric stretching (υ_1_ PO_4_) while less intense bands at ∼608 and 565 cm^−1^ are due to the (υ_4_ PO_4_) bending mode of ${\text{PO}}_{4}^{3-}$ groups [17, 19]. The presence of carbonate (${\text{CO}}_{3}^{2-}$) bands is attested by vibrational signatures due to the υ_3_CO_3_ mode, with maxima around ∼1415 and 1473 cm^−1^, and the υ_2_ CO_3_ mode with a peak around 873 cm^−1^. Detailed analysis of the υ_2_CO_3_ region shows the signals at ∼879 and 872 cm^−1^ corresponding to A-type and B-type carbonate substitutions, with carbonate ions replacing respectively OH^−^ and ${\text{PO}}_{4}^{3}$^−^ lattice ions. Beside apatitic vibrational contributions, a band at ∼1590 cm^−1^ is also noticed on all samples, which can be ascribed to the antisymmetric stretching frequencies of the carboxylate groups of the citrate. Bands at ∼2930 cm^−1^ (not shown) and ∼840 cm^−1^ (shoulder) are assignable to υCH_2_ and δCOO modes of the citrate ions [20], respectively.

The presence of carbonate substituting –OH and ${\text{PO}}_{4}^{3}$^−^ lattice ions in the crystal structure as well as of a citrate layer coating the surface of the nano-HAp crystals is also a characteristic feature of the bone apatite. In bone, the apatite component is poorly crystalline and also displays a plate-like morphology and is doped with carbonate [21]. Recently, Hu *et al*. [22] have revealed that citrate, which accounts for about 5.5 wt % of total organic matrix of bone, is found strongly bound to the surface of the apatite nanoparticles. The use of carbonate and citrate in the mother solutions is thus, a straightforward nature inspired strategy to prepare bonelike apatites to be used in combination with the new hydrogels for the tissue regeneration.

### Microspheres and collagen based-hydrogel characteristics

**Figure 2:**
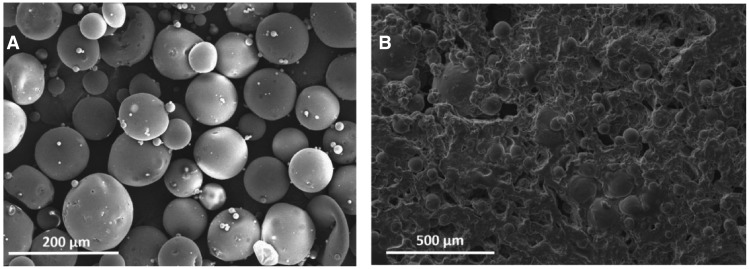
(A) SEM image of lyophilized PLGA microspheres. (B) SEM image showing the internal structure of hydrogel

The mean diameters in volume of the RITC-dextran-loaded PLGA microspheres and β-estradiol-loaded PLA/PLGA microspheres were 99.70 ± 5.00 µm and 119.14 ± 0.25 µm, respectively. Both PLGA and PLA/PLGA microparticles were generated using PVA which is adsorbed in the surface of the particle and protect them from agglomeration. Shakesheff *et al*. [23] indicated that part of PVA molecules remain in the surface of the particles after consecutive washing and cannot be removed since PVA forms a stable network with PLGA. Figure 2A shows a SEM image of the generated PLGA microspheres. The encapsulation efficiency of RITC-dextran and β-estradiol in the microspheres was 80.1 ± 7.4% and 74.8 ± 5.2%, respectively. The high porosity of dry hydrogel, 81.62 ± 2.25%, was decreased up to 69.98 ± 3.07% when microspheres and nano-HAp were incorporated. Figure 2B shows a SEM image of the porous internal structure of the hydrogel after the incorporation of microspheres.

PLGA and PLA/PLGA microspheres were generated without the use of a porogen to avoid a rapid release of the model drugs. Oh *et al*. [24] showed a decrease in encapsulation efficiency and a large increase in the release rate of budesonide when ammonium bicarbonate (porogen) was included to generate PLGA microspheres. As can be seen in the SEM image (Fig. 2A), the surface of microspheres is solid and flat and no pores can be seen. Zhang *et al*. [25] reported the synthesis of porous and non-porous PLGA microspheres by the simple emulsion solvent evaporation method. In the absence of a porogen, no pores were found in the resultant particles that had a smooth surface; however, by using 2-methylpentane in the oil phase as porogen, porous particles were generated. In the case of the technique of double emulsion solvent evaporation, several authors have demonstrated the generation of porous PLGA microspheres by the addition of ammonium bicarbonate [24, 26] or phosphate buffered saline [27] in the first aqueous phase. In the absence of a porogen, these authors did not found pores in the particles that showed a smooth surface.

In the other hand, citrate-coated carbonated apatite nanoparticles prepared by thermal decomplexing are non-porous and tend to aggregate with time in mesoporous materials with a mean pore diameter in the range between 100 and 200 Å [16].

The inclusion of non-porous microparticles and mesoporous nano-HAp to the highly porous hydrogel will lead to a reduction in their porosity. In fact, Wang *et al*. [26] showed the reduction of porosity of sintered scaffolds by the inclusion of non-porous PLGA microspheres (from 87.50 ± 5.23% for scaffolds including porous particles generated with a 1:10 porogen–polymer ratio to 43.2 ± 9.39% for scaffolds including non-porous particles).

### Viscoelastic behavior and interactions of compounds on hydrogel

**Figure 3:**
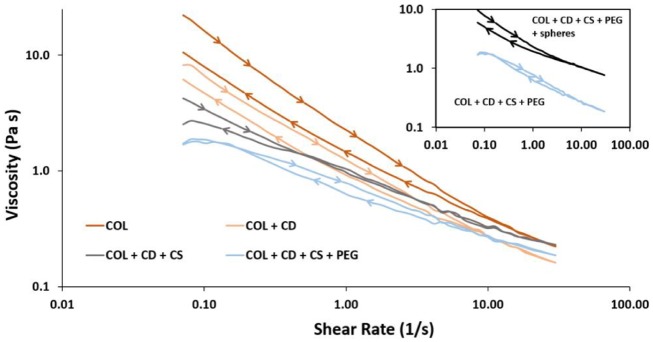
Variation of viscosity of collagen solution (10 mg/ml), before (COL) and after cyclodextrin (COL+CD), chitosan (COL+CD+CS) and PEG (COL+CD+CS+PEG) incorporation, with shear rate at 37°C. The upper box shows the viscosity versus shear rate of the complete gel, before (COL+CD+CS+PEG) and after the incorporation of microspheres and nano-hydroxyapatite (COL+CD+CS+PEG + spheres)

The incorporation of cyclodextrin, chitosan and PEG to the initial collagen solution (10 mg/ml) (COL) did not add additional viscosity, but generated consecutive dilutions as can be seen in Fig. 3, where viscosity versus shear rate at 37°C is represented. As expected, the incorporation of microspheres and nano-hydroxyapatite produced an increase in the viscosity values (Fig. 3, top right box).

Collagen, as many other polymers with long chains, has a non-Newtonian pseudoplastic behavior, i.e. viscosity decreases as the shear rate increases, being this process reversible when the stress is removed. In particular, collagen solutions have thixotropic properties, taking some time to recover the initial viscosity values after stress have been removed since the rearrangement of its structure is a time-dependent process. In Fig. 3, the values of viscosity after the application (forward curve) and removal (backward curve) of stress did not match, mainly for the collagen solution (COL) and the complete gel with spheres (COL+CD+CS+PEG + spheres). The forward and backward curves did not overlap, being the area between both (or hysteresis loop) an indication of the magnitude of the thixotropic behavior that permit the implantation of hydrogel in a minimally invasive way and facilitate the adaptation to the shape of the area to be treated, before being crosslinked ‘in-situ’ by means of blue light and TPP.

The different interactions between the components of the hydrogel: collagen, cyclodextrin, chitosan and PEG, generate a gel network i.e. able to trap the bioactive components, i.e. the pre-encapsulated bioactive substances and nano-hydroxyapatite.

**Figure 4:**
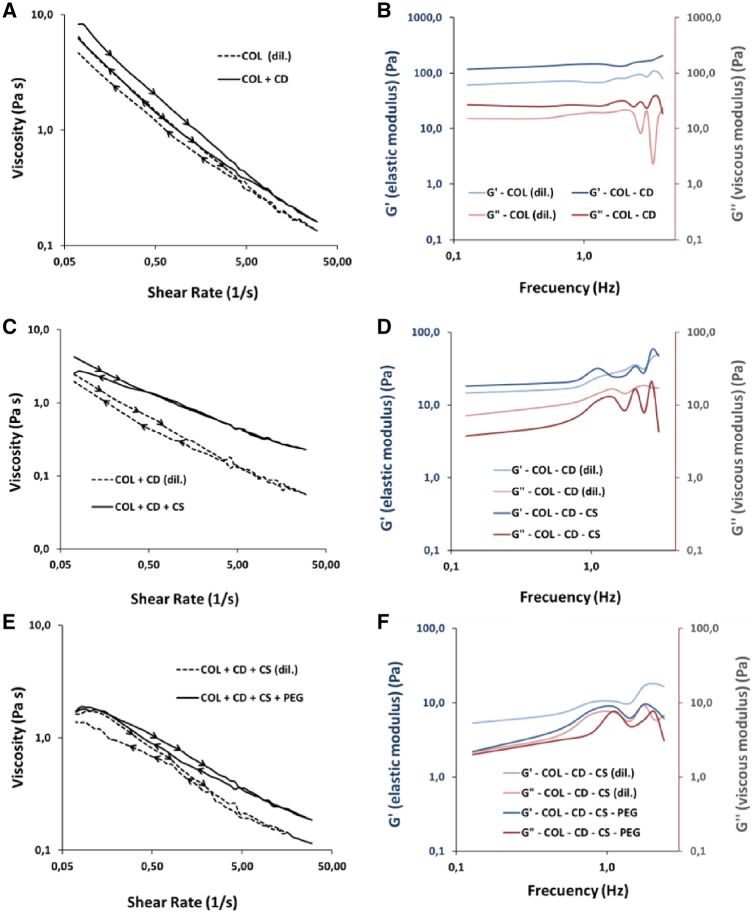
Evolution of the viscoelastic behavior of collagen with the incorporation of cyclodextrin, chitosan and PEG. Variation of (A) viscosity with shear rate and (B) elastic and viscous moduli with frequency in collagen (COL) versus a mixture of collagen and cyclodextrin (COL+CD) with a mass ratio equal to that in the final gel, maintaining the collagen concentration in both samples (COL) and (COL+CD). Variation of (C) viscosity with shear rate and (D) elastic and viscous moduli with frequency in collagen–cyclodextrin mixture (COL+CD) versus a collagen–cyclodextrin–chitosan mixture (COL+CD+CS) with a mass ratio equal to that in the final gel, maintaining the collagen and cyclodextrin concentration in both samples (COL+CD) and (COL+CD+CS). Variation of (E) viscosity with shear rate and (F) elastic and viscous moduli with frequency in collagen–cyclodextrin–chitosan mixture (COL+CD+CS) versus the complete gel (COL+CD+CS+PEG) with a mass ratio equal to that in the final gel, maintaining the collagen, cyclodextrin and chitosan concentrations in both samples (COL+CD+CS) and (COL+CD+CS+PEG)

Collagen is a protein with a special and atypical combination of amino acids, being glycine (Gly), proline (Pro) and hydroxyproline (Hyp) the most repeated units that are responsible of the triple-helical structure. Glycine, the dominant unit in collagen, facilitates the formation and stabilization of collagen structure by internal hydrogen bonding. This amino acid can be mostly part of the sequences, Gly–Pro–X or Gly–X–Hyp, where X can be any other amino acid with different functional groups, like carboxyl and amino groups, in the outside part of the helix that are available for reaction with other molecules [28, 29]. At the very end of both sides of collagen molecules, there are terminal carboxyl and amino groups that are not incorporated in the triple-helix and are part of two non-helical domains (telopeptides) that are available for reaction with other molecules [30]. The existence of hydrophobic residues within the telopeptides region may allow their incorporation to the hydrophobic cavities of cyclodextrins, compromising the crosslinking process in collagen and the subsequent fibrillogenesis and aggregation processes [30]. In fact, cyclodextrin is a fundamental component in the hydrogel by allowing the incorporation of hydrophobic compounds, like the β-estradiol, directly in the gel without the need to pre-encapsulate them in spheres. Moreover, cyclodextrin can reinforce the collagen matrix by means of hydrogen bonds between the multiple hydroxyl groups of cyclodextrin and the terminal and/or side hydroxyl, amino, amide or carboxyl groups of collagen. Figure 4A and B represents the variation of viscosity with the shear rate and the elastic and viscous modulus with the frequency, respectively, for a collagen solution (COL) and a mixture of collagen and cyclodextrin (COL+CD). These figures demonstrated the above-mentioned interactions between collagen and cyclodextrin. In this regard, the incorporation of cyclodextrin to the collagen solution increases the viscosity values (Fig. 4A) and the solid behavior of collagen (both modulus increase, although the elastic to a greater extent, staying above in both cases) (Fig. 4B). Note that the same mass ratio of the complete gel and the same collagen concentration was maintained to avoid the dilution effect that was commented above (Fig. 3).

**Figure 5:**
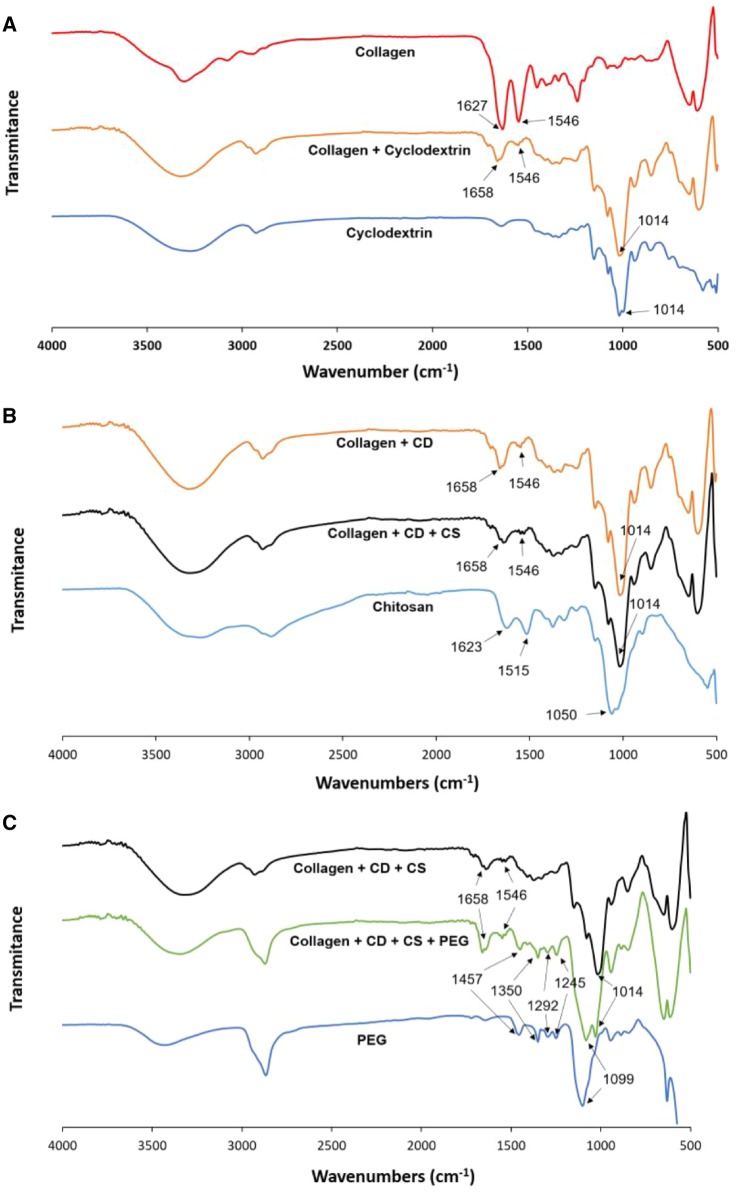
FT-IR spectra in transmittance mode for (A) collagen, cyclodextrin and the mixture of both; (B) chitosan, collagen/cyclodextrin and collagen/cyclodextrin/chitosan mixtures; (C) PEG, collagen/cyclodextrin/chitosan and collagen/cyclodextrin/chitosan/PEG mixtures

The existence of hydrogen bonds between cyclodextrin and collagen is observed in Fig. 5A, from the comparison of the fingerprint region of the FT-IR spectra (from 1700 to 500 cm^−1^) of collagen solution and collagen/cyclodextrin mixture. The collagen spectra shows the typical bands at 1627 cm^−1^ (amide I: C = O stretching) and 1546 cm^−1^ (amide II: N–H deformation and C–N stretching). The incorporation of cyclodextrin to collagen produces a shift in the amide I band to higher wave numbers (1658 cm^−1^) and a great decrease in the intensity of the amide II band (1546 cm^−1^) in comparison with the amide I band that may suggest the formation of new hydrogen bonds between peptide carbonyl groups in collagen and hydroxyl groups in cyclodextrin. Note that the amide II band is not present in the cyclodextrin spectra. The appearance of the band at 1014 cm^−1^ is due to the C–O vibrations because of the contribution of ether and hydroxyl groups of cyclodextrin molecules to the mixture as can be seen in the cyclodextrin spectra of Fig. 5A.

Chitosan was added to the collagen–cyclodextrin mixture to harden the hydrogel by crosslinking with TPP. Hydroxyl and amino groups in chitosan are able to form hydrogen bonds with side groups or carboxyl and amino end groups of collagen. Ionic interactions between the positively charged amino groups in chitosan and the negatively charged carboxyl end groups in the telopeptide areas of collagen are also possible [31]. Because of these interactions, chitosan may be rolled around triple-helix of collagen, forming a complex with an increased viscosity compared to the collagen–cyclodextrin mixture. This behavior can be seen in the evolution of viscosity with shear rate of collagen–cyclodextrin (COL+CD) and collagen–cyclodextrin–chitosan (COL+CD+CS) mixtures (Fig. 4C). Moreover, the incorporation of chitosan (COL+CD+CS) increase the elastic behavior of the collagen–cyclodextrin mixture (COL+CD) since the difference between elastic and viscous modulus becomes bigger (Fig. 4D). Note that the comparison between COL+CD and COL+CD+CS was performed at the same conditions as Fig. 4A and B.

The incorporation of chitosan to the collagen/cyclodextrin mixture did not produce major changes in the fingerprint region of the IR-spectra (Fig. 5B), showing the amide I band at 1658 cm^−1^, the amide II band of much lower intensity at 1546 cm^−1^ and the C–O band at 1014 cm^−1^. This absence of changes in the IR-spectra indicates the predominance of the ionic interactions between the cationic amino groups in chitosan and the anionic carboxylic end groups in collagen against the hydrogen bonds between both molecules.

Chitosan spectra in the fingerprint region (Fig. 5B) showed mainly the amide I band at 1623 cm^−1^ and the C–O stretching at 1050 cm^−1^. This spectra also shows a band at 1515 cm^−1^ that may be due to the N–H bending of primary amines, since its greater intensity with respect to the amide I band do not match with the amide II band, which is not present in this spectra.

The last component to be incorporated to hydrogel was PEG that may produce the molecular crowding of collagen, forming aligned fibrils. However, minor reversible interactions between collagen and PEG by hydrogen bonding and ionic interactions may be also possible [32, 33]. These facts may be supported by the increase on viscosity in all the range of shear rate (Fig. 4E), when PEG is included to the collagen–cyclodextrin–chitosan mixture. At low frequencies, the big difference between the elastic and viscous modulus of the collagen–cyclodextrin–chitosan mixture is decreased when the PEG is incorporated, as shown in Fig. 4F. This behavior may be caused by an approach to a Newtonian behavior because of the great dilution of the final mixture, but, despite this, the hydrogel remains elastic in the complete gel since the elastic module is above the viscous module in all the frequency range. Note that comparison between COL+CD+CS and COL+CD+CS+PEG were generated at the same conditions as Fig. 4A and B. Although it is well known the formation of inclusion complexes (polyrotaxanes) between alpha-cyclodextrin and PEG [34, 35], the utilization of beta or gamma cyclodextrin as an alternative of alpha-cyclodextrin avoid the formation of polyrotaxane structures [36].

PEG 400 IR-spectra in the fingerprint region (Fig. 5C) shows the C–H bands at 1457 and 1350 cm^−1^, the C–O bands corresponding to alcohol at 1292 and 1245 cm^−1^, and the C–O–C of ether at 1099 cm^−1^. The incorporation of PEG 400 to the hydrogel did not alter the previously commented IR-spectra of collagen/cyclodextrin/chitosan mixture, beyond the superimposition of both spectra. Only a small increase in the difference between the intensities of the amide I and amide II peaks (Fig. 5C) can be observed. This small change may indicate a higher probability of occurrence of hydrogen bindings and ionic interactions between collagen and PEG over the molecular crowding of collagen.

### *In vitro* release profiles

**Figure 6:**
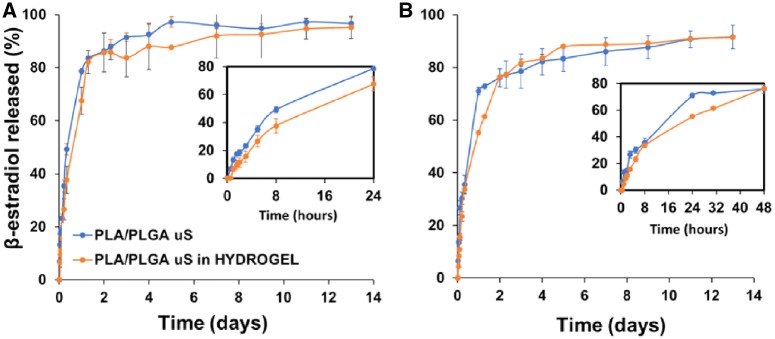
*In vitro* release profile of β-estradiol from microspheres and microspheres-loaded hydrogel. In the upper right part of the figure is shown a magnification of the first hours. (A) Semi-continuous sampling method; (B) batch sampling method

**Figure 7:**
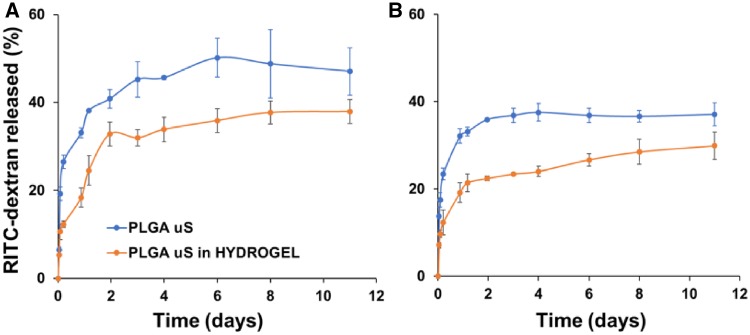
*In vitro* and *in vivo* release profiles of RITC-dextran from microspheres and microspheres-loaded hydrogel. (A) Semi-continuous sampling method; (B) batch sampling method

*In vitro* release profiles of β-estradiol (Fig. 6) or RITC-dextran (Fig. 7) from the microsphere-loaded hydrogel reveal a two-phase behavior with a burst and a controlled release period. By comparing these results with the respective profiles of microspheres, it is possible to conclude that burst release was reduced (see enlargement of Fig. 6 or 7) because of the different interactions between the components of the hydrogel previously mentioned and the crosslinking process that entrap the bioactive substances. Burst release for β-estradiol is accentuated during the first 24 h, while for RITC-dextran is moderate the first 2–3 days, being the damping effect of the hydrogel much more visible in the case of the latter. The controlled release period is produced the following 15 days or 3 weeks for β-estradiol or RITC-dextran, respectively. The fact that this period occurs at the same rate for the microspheres included or not in the hydrogel may indicate that the release process is mainly controlled by internal diffusion within microspheres. However, depending on the sampling method, the final released fraction varies. When media is returned after the measurement (Fig. 6B or 7B), the drug is accumulated continuously and the total drug released before the process stops will be less than in the case of removing the sample after the measurement (Fig. 6A or 7A). This is because in the latter case, the media is renewed continuously and the concentration gradient between the spheres and the media is increased each sampling time, which forces the remaining drug to be released from the spheres until the concentration inside them is at equilibrium with the media.

Experiments were carried out under sink conditions for both, hydrophobic and hydrophilic model drugs. Thus, β-estradiol was almost completely released from the spheres, i.e. 97 or 92% for the semi-continuous or batch sampling methods, respectively (Fig. 6), being able to assure that there was not crystallization inside the particles. However, not all the RITC-dextran molecules diffuse out and the final released fraction was in the range between 29 and 47% for all the experiments with this molecule (Fig. 7). These data are in accordance with the literature [37–39] and may be due to electrostatic interactions between the negatively charged deprotonated carboxyl groups of PLGA and the positively charged amine groups of rhodamine from the RITC-dextran at pH values between 6 and 7 [40]. The complete release of this model drug can be achieved at acidic pH values of 4–5 [40]. Note that the slower release rate of RITC-dextran with respect to the data from literature is due to the high molecular weight of dextran.

### Mass transfer studies for hydrophobic and hydrophilic drugs

The mass transfer process that occurs from polymeric microspheres, as isolated systems or included in porous hydrogels, to the media in body cavities with low clearance rates was simulated using two model compounds, 17-β-estradiol and RITC-dextran, which mimic low molecular weight hydrophobic and high molecular weight hydrophilic drugs, respectively.

Some systems based on natural polymers, like alginate or chitosan, with short release times [41–43] behave as concentrated systems, i.e. uniform drug concentration within microspheres. Then, diffusional resistance inside the microspheres is negligible compared to the liquid boundary layer resistance i.e. the rate-limiting step, being able to describe the simplified release process by means of the mass transfer coefficient, *h* [41, 42]. However, based on the experimental *in vitro* profiles mentioned above, a distributed system must be assumed, where the concentration gradient inside the microspheres is rate limiting. Thus, the release process is mainly described by means of the diffusion process inside the spheres and therefore by the effective diffusion coefficient, *D*_eff_ [44, 45].

Diffusion process from the spherical microparticles is governed by Fick’s second law of diffusion in radial coordinates [44], according to the Equation (1):

(1) $$\frac{\partial C}{\partial t}=D_{\mathrm{eff}}\left(\frac{\partial^{2}C}{\partial r^{2}}+\frac{2}{r}\frac{\partial C}{\partial r}\right)\;\mathrm{for}\;0<r<R,\;$$

where *C* is the concentration of the drug, *D*_eff_ is the effective diffusion coefficient, *t* is the time, *r* is the radial coordinate and *R* is the radius of the microparticles.

Equation (1) is solved with the surface boundary condition of no accumulation described by Equation (2), which assumes that the mass flow rate of the drug i.e. released to the media is equal to the mass flow rate of the drug that reach the surface of the spheres by diffusion [44, 45]:

(2) $$-D_{\mathrm{eff}}\left(\frac{\partial C}{\partial r}\right)=h\left(C_{S,\mathrm{sf}}-C_{0,\infty }\right)\;\mathrm{for}\;r=R,\;$$

where *C_S_,*_*sf*_ is the drug concentration inside the spheres, but in the surface; *C*_0,__*∞*_ is the drug concentration in equilibrium with the liquid; *D*_eff_ is the effective diffusion coefficient; *r* is the radial coordinate; *R* is the radius of the microparticles and *h* is the mass transfer coefficient in the boundary layer.

Resolution of Equation (1) with Equation (2) for a spherical geometry, considering a homogeneous distribution of the drug inside the spheres at the beginning of the process, i.e. *C(r)* *=* *C*_0_*at t = 0,* for *0 < r < R* (monolithic system), leads to Equation (3). This expression permits the calculation of the fraction of the cumulative amount of drug leaving the microspheres as a function of time [44, 45]. Note that, *C*_0_ is the initial drug concentration inside the spheres, and perfect sink conditions during all the experiments were assured.

(3) $$\frac{M_{t}}{M_{\infty }}=1-\sum_{n=1}^{\infty }\frac{6\;L^{2}}{{\beta_{n}}^{2}\;\left({\beta_{n}}^{2}+L^{2}-L\right)}e\mathrm{xp}\left(-\frac{{\beta_{n}}^{2}}{R^{2}}\;D_{\mathrm{eff}}\;t\right),$$

where *M_t_* and *M_∞_* are the total mass of drug released to the media at time *t* and at the final of the process, respectively. *M_∞_* can be obtained from the concentration of the drug inside the spheres at the beginning of the process (*t = 0*) i.e. the value obtained by dissolution of the spheres. The β*_n_s* are the infinite roots (eigenvalues) of the Equation (4):

(4) $$\beta_{n}\cot\beta_{n}+L-1=0.\;$$

*L* is the dimensionless mass transfer Biot number, described by the Equation (5):

(5) $$L=\frac{h\;R}{D_{\mathrm{eff}}}\;.$$

For *L* below 0.01, the drug concentration within microspheres is uniform, so the liquid boundary layer surrounding the spheres is rate limiting and the spheres behaves as a concentrated system; while for *L* above 10 the spheres acts as a distributed system, being the resistance inside the polymer matrix of the spheres rate limiting.

For large values of *L*, the roots of Equation (4) are multiple of the number *pi* and Equation (3) can be simplified in Equation (6) i.e. a simplified solution of Fick’s second law of diffusion:

(6) $$\frac{M_{t}}{M_{\infty }}=1-\frac{6}{\pi^{2}}\sum_{n=1}^{\infty }\frac{1}{n^{2}}e\mathrm{xp}\left(-\frac{{n^{2}\;\pi }^{2}}{R^{2}}\;D_{\mathrm{eff}}\;t\right).\;$$

Since microspheres in all cases are assumed as distributed systems, they must contribute in the same way to the global mass transport process, whether or not they are included in the hydrogel. Thus, the latter can be considered as part of the boundary layer between the spheres and the media. Equations (3–6) describe the complete mass transfer process, permitting to determine not only the effective diffusion coefficient in the polymer matrix of the spheres, *D*_eff_, but also the mass transfer coefficient in the boundary layer, *h*, to which the hydrogel contributes to a greater extent.

Table 1 shows, for each sampling method, batch and semi-continuous, the values of *D*_eff_ that describe the internal diffusion of 17-β-estradiol and RITC-dextran inside microspheres and are in the range of 2.64·10^−15^ to 6.67·10^−15^ m^2^/s. The contribution of hydrogel to the mass transfer process throughout the values of *h*, which are in the range of 8.50·10^−10^ to 3.04·10^−9^ m^2^/s, are also shown in Table 1. The behavior of both model drugs was similar in the system regardless of the sampling method and no tendency was detected.

**Table 1:** Effective diffusion coefficient, *D_e_* (m^2^/s), and mass transfer coefficient, *h* (m/s), values for 17-β-estradiol and RITC-dextran according to Equations (3–6)

Sampling method	*D_e_* (m^2^/s)		*h* (m/s)		*R* ^2^ (%)/RSS values	
	β-Estradiol	RITC-dextran	β-Estradiol	RITC-dextran	β-Estradiol	RITC-dextran
Batch	2.9281·10^−15^	6.6716·10^−15^	8.5030·10^−10^	3.0350·10^−9^	98.87/1.2857·10^−3^	95.41/4.4388·10^−4^
Semi-continuous	3.9852·10^−15^	2.6409·10^−15^	9.2152·10^−10^	1.2050·10^−9^	98.29/2.2671·10^−3^	95.72/8.6032·10^−4^

**Figure 8:**
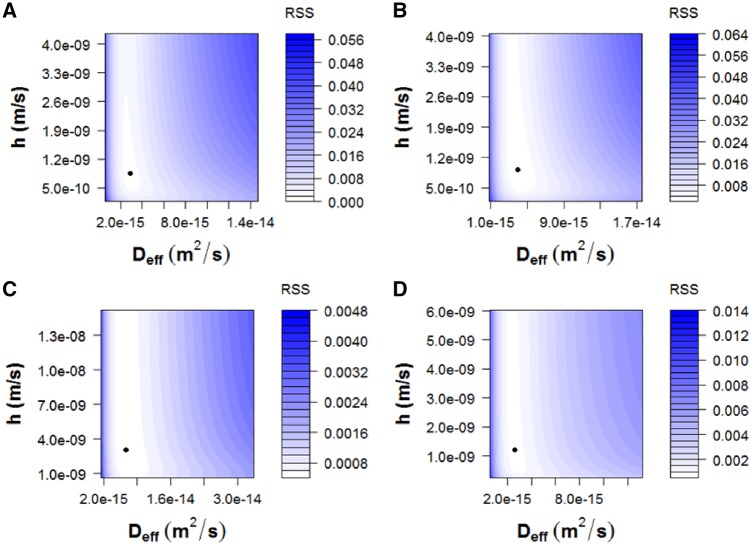
Location of the predicted *D*_eff_ and *h* within the residual sum of squares (RSS) level curves. (A) 17-β-Estradiol, batch sampling method; (B) 17-β-estradiol, semi-continuous sampling method; (C) RITC-dextran, batch sampling method; (D) RITC-dextran, semi-continuous sampling method

**Figure 9:**
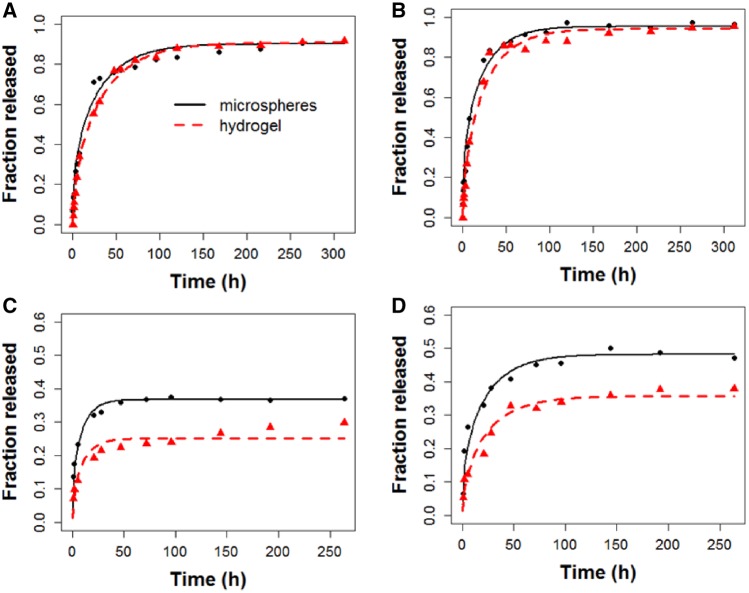
Comparison of the experimental released fractions, *M/M*_∞_ (points) versus the simulated ones (lines) for: (A) 17-β-estradiol, batch sampling method; (B) 17-β-estradiol, semi-continuous sampling method; (C) RITC-dextran, batch sampling method; (D) RITC-dextran, semi-continuous sampling method

To obtain *D*_eff_ and *h*, deviations between experimental released fractions, *M/M_∞_*, from microspheres included or not in hydrogels and the predicted *M/M_∞_* according to Equations (3–6) were minimized. This was made for each model drug and sampling method with ‘genetic algorithms’, already implemented in ‘R software’ (R Foundation for Statistical Computing, version 3.5.1., 2018, Vienna) [46, 47], because of the ill-conditioned nature of diffusion equation. The ‘residual sum of squares (RSS)’ values obtained were between 4.44·10^−4^ and 2.27·10^−3^ and the *R*^2^-values were ranged between 95.41 and 98.87%. The mathematical form of diffusion models is an infinite series of terms itself nonlinear, making difficult the use of the classical methods to fit nonlinear models. The RSS allows identifying two common problems: multiple minima and strong nonlinear profiles, which can result in a relative minimum, not the absolute one. Note that predicted *M/M_∞_* was obtained using Equation (6) for microspheres and Equations (3–5) for microspheres included in hydrogels. The value of *D*_eff_ was the same for microspheres, whether they were included or not in the hydrogel, for each condition. The hydrogel contributed to the mass transfer process with *h*, as part of the boundary layer. The location of the predicted *D*_eff_ and *h* within the RSS level curves, obtained for a great variety of values of both coefficients (Fig. 8), and the comparison (Fig. 9) of the experimental released fractions, *M/M_∞_*, (points) versus the simulated ones (lines) demonstrate that the proposed model fits the experimental data for both model drugs.

### *In vitro* cell culture in hydrogels

**Figure 10:**
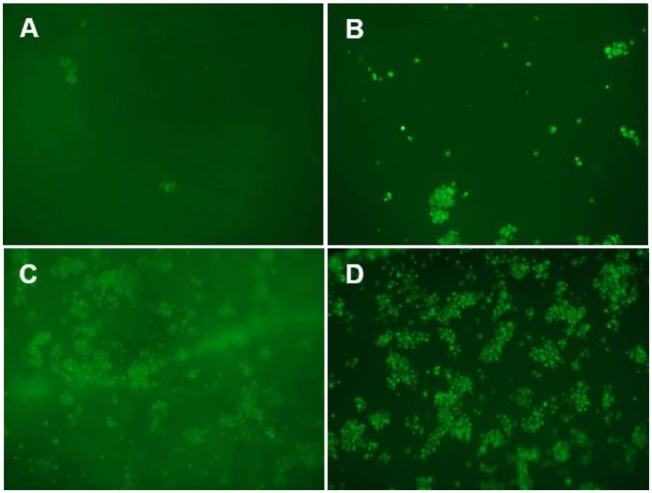
rMSC seeded on the hydrogel scaffold. Representative photomicrographs (10×) of cell viability by calcein-AM staining after (A) 1.5 h (time for cellular adhesion); (B) 1 day; (C) 3 days and (D) 7 days of incubation in complete culture medium at 37°C and 5% CO_2_

The hydrogel presented adequate characteristics for a good cell adhesion, viability and proliferation. The green fluorescent intensity produced by the living cells was increased over the time (Fig. 10). At 1.5 h a few cells were observed on the surface of the scaffold. The cell proliferation increased and after 7 days, most of the scaffold was occupied by living cells.

## Conclusions

An innovative thixotropic hydrogel based on chitosan, collagen, HP-γ-CD and PEG has been developed. In order to target its use in bone regeneration, the hydrogel incorporated nano-hydroxyapatite and microspheres loaded with hydrophobic or hydrophilic model drugs (17-β-estradiol or RITC-dextran) that slightly diminished the porosity of hydrogel to 69.98 ± 3.07%.

The changes in the FT-IR spectra, the increase of viscosity and the intensification of the solid behavior of collagen with the incorporation of cyclodextrin suggested the incorporation of the residues of the telopeptide region of collagen inside the cavities of cyclodextrins and the formation of new hydrogen bonds between both molecules. In a similar way, the rheological parameters indicated the formation of hydrogen bonds between chitosan and collagen; however, the FTIR spectroscopy suggested the predominance of ionic interactions over the hydrogen bonds between both molecules. The increase on viscosity, the maintenance of the elastic behavior and the lack of variation in FT-IR spectra when PEG was incorporated to the rest of components, suggested the existence of ionic interactions between collagen and PEG. The interactions between components together with the rheological behavior of hydrogel explain the formation of a gel network that allows to trap the bioactive components and facilitate the adaptation and injection of the hydrogel to diverse treatment sites.

*In vitro* release profiles of pre-encapsulated model drugs showed a two-phase behavior with a burst release period that was damped by hydrogel and a controlled release period with a similar rate whether they are included or not in hydrogel, which indicated a mass transfer process controlled by internal diffusion within microspheres. Thus, a distributed system was assumed to model mass transfer process of drugs from polymeric microspheres, as isolated systems or included in porous hydrogels, to the media in body cavities with low clearance rates. The calculated effective diffusion coefficients, *D*_eff_, that describe the internal diffusion of the model drugs inside the microspheres, and the mass transfer coefficients, *h*, i.e. the contribution of hydrogel to the mass transfer process, as part of the boundary layer, did not show any tendency regardless of the sampling method or the model drug used.

The *in vitro* culture of rMSC in hydrogel showed no signs of intolerance or toxicity, observing an intense proliferation of the cells after 7 days, being most of the scaffold surface occupied by living cells.

Since mass transfer process is controlled by the internal diffusion within microspheres, in order to obtain an optimal release profile, an improvement in the structure of the microspheres would be necessary. To do that, new combinations of polymers to form their matrix or different type of coatings should be tested. Moreover, as long as the hydrogel continues to be a good support for bioactive substances and presents good cell adhesion, viability and proliferation properties, a greater contribution of the hydrogel in the mass transfer process would be desirable.
